# Manual handling of heavy loads and low back pain among different occupational groups: results of the 2018 BIBB/BAuA employment survey

**DOI:** 10.1186/s12891-021-04819-z

**Published:** 2021-11-15

**Authors:** Martha Sauter, Julia Barthelme, Charlotte Müller, Falk Liebers

**Affiliations:** 1 Charité-Universitaetsmedizin Berlin, Berlin, Germany; 2 Federal Institute for Occupational Safety and Health (BAuA), Division “Work and Health” / unit 3.1 “Prevention of Work-Related Disorders“, Noeldnerstrasse 40-42, 10317 Berlin, Germany

**Keywords:** Working conditions, BIBB/BAuA employment survey, Gender; prevalence, Musculoskeletal system, Employment, Occupations, Health status, Workforce

## Abstract

**Background:**

In Germany and other European countries, many occupations still involve manual handling of loads (MHL), an activity that puts the musculoskeletal system at risk of low back pain (LBP). This study aims to describe the current prevalence of MHL in different occupational groups stratified by gender in Germany, the association between MHL and LBP and the adjusted prevalence of LBP in different respond-categories of MHL.

**Methods:**

Data was collected in telephone interviews conducted as part of the 2018 BIBB/BAuA Employment Survey, which covers work-related topics like working conditions, education, health status and job satisfaction. The analyses were limited to full-time workers (> 35 h/week) aged between 15 and 67. The frequency of MHL was analysed descriptively. BLOSSFELD classification was used to group the participants in occupational categories. The analysis of the association between MHL and the prevalence of LBP over the last 12 months was based on robust log-linear Poisson regression that results in prevalence ratios (PR). The main regression model was adjusted for gender, age, working hours, and working conditions. Adjusted estimates for the prevalence of LBP were calculated based on regression analysis.

**Results:**

The sample consists of *n* = 14,331 participants (men: *n* = 8828, 61.6%; women: *n* = 5503, 38.4%; median age 49 years). Of these, 52.8% say they were exposed to MHL at work. MHL is most common in agricultural occupations, skilled and unskilled occupations. In the regression model, participants who said they were “often” exposed to MHL reported more frequently LBP than those participants who said they were “never” exposed to MHL. The PR as estimate for the association is 1.41 (95%CI [1.32; 1.49]). Postestimation of the prevalence of LBP began with 47.3% (95%CI [43.8%; 51.1%]) for participants who said they were “never” exposed to MHL and rose to 66.5% (95%CI [62.4%; 71.0%]) for participants who indicated they were “often” exposed to MHL.

**Conclusions:**

The 2018 BIBB/BAuA Employment Survey emphasizes that MHL is still common in the German workforce and shows a significant association to LBP. Prevention policies for avoiding MHL remain vital.

**Supplementary Information:**

The online version contains supplementary material available at 10.1186/s12891-021-04819-z.

## Background

Manual handling of loads (MHL) is still a common physical workload at workplaces in Germany. According to the 2012 BIBB/BAuA Employment Survey, one fourth of employees said they “often” manually handled loads. Half of these respondents also said they suffered under this working condition [1]. Strain occurs during the packing and unloading of containers as well as when transporting furniture or during patient handling [2]. The European Agency of Safety and Health at Work (EU-OSHA) reported a proportion of 32 to 35% of workers who carried or moved heavy loads for at least a quarter of their working time across the EU28 states between 2005 and 2015 [3]. To protect employees from its adverse health consequences, MHL should be avoided as far as possible [4].

MHL is well known to be a risk factor for back pain [5, 6], which causes a major share of health costs in Germany and is one of the main reasons for incapacity for work [7, 8]; it also negatively affects the quality of life and everyday activities [9–11]. According to the German Federal Statistical Office, 3.2% of total health-care-costs in 2015 were caused by dorsopathies (ICD-10: M45 –M54) [7]. An annual data report of one of the German health insurances showed that, in 2017 back pain (ICD-10: M54) was the cause of 5.8% of sick days [8]. There is evidence that further work-related, physical and climatic factors such as heavy physical work, awkward postures, whole-body-vibrations, slipping and falling or working in cold environments contribute to the multifactorial causes of back pain [6, 9, 12, 13]. Furthermore, sitting and standing a have also been shown to be associated with low back pain (LBP) [14–16]. Apart from these physical working exposures, psychosocial factors and working hours also affect the genesis of musculoskeletal disorders [6, 17, 18]. Gender, age, anthropometric characteristics, socioeconomic status, and smoking are other factors that influence back pain [5, 6, 9, 19].

Conducting risk assessments at the workplace is considered a basic step to derive and implement effective measures of primary prevention to reduce physical workload as a work-related risk factor of low back pain [20]. In a work-specific context, this strategy has also been embedded in German and European legislation [21, 22]. Even though digitalization and technological progress are changing working environments, resulting in a decrease in physical workload, there are still professions in which physical strains are high [23].

It is important to use existing data to keep MHL under surveillance in Germany and to contribute to European surveillance. According to Hulshof et al. (2021) [24], only few studies present current data. Furthermore, the results of our analysis support the preparation and justification of preventive measures in the third period of the Joint German Occupational Safety and Health Strategy (“GDA”, www.gda-portal.de) regarding the prevention of musculoskeletal workloads at workplaces. By determining the current prevalence of MHL in different professions, target-oriented primary prevention programs can be implemented. Reducing the prevalence of MHL – as a risk factor for low back pain – can help to reduce the prevalence of low back pain as one of the most expensive disorders in workers with high physical demands [7, 8, 13].

This study aims to investigate the current prevalence of manual handling of heavy loads in different occupational groups stratified by gender in Germany, the association between MHL and self-reported LBP, and adjusted prevalence of low back pain in the different respond categories of MHL, using the 2018 BIBB/BAuA Employment Survey conducted by the Federal Institute of Occupational Training (BIBB) and the Federal Institute for Occupational Safety and Health (BAuA) [25]. The STROBE checklist was used to secure transparent reporting in this paper [26].

## Methods

### Study design and setting

The 2018 BIBB/BAuA Employment Survey [25] is an interview-based, cross-sectional study. The survey is conducted periodically every 6 years and aims at gathering information about the working conditions of the German workforce. All participants gave their verbal consent during the telephone interview. The BAuA ethics committee approved the study and its procedure (EK007_2017 January 9, 2017). Interviews of 20,012 employees were conducted by the social research company Kantar Public from August 2017 to April 2018 using a computer-assisted telephone questionnaire. For the sampling a random-digital-dialing approach was used. For the landline numbers, a two-stage process, so called Kish-Selection-Grid, was implemented to secure equal chances to get interviewed. For mobile numbers it was assumed that the devices are used only by one person. To minimize selection bias, a dual frame approach was implemented to enable interviewers to also contact persons who are only available via cell phone. Furthermore, participants were interviewed in the afternoon, in the evening and on weekends. This led to a sample that consists of 70% landline and 30% mobile network users. Interviewers needed 40 min on average to complete the questionnaire in full. The questionnaire was developed based on the previous 2012 BIBB/BAuA Employment Survey. The main parts are identical to this survey, but some questions were supplemented by new questions. The main topics of the survey, which were assessed are information on the respondent’s current occupation, working conditions, education, health status and job satisfaction [27]. Further information about the methods of the 2018 BIBB/BAuA Employment Survey are available online (www.bibb.de) [25].

### Participants and study size

Participants were included in the 2018 BIBB/BAuA Employment Survey if they were 15 years of age and over, were employed for 10 h per week or more and had an adequate command of the German language. It was conditional to be paid for their work to meet the selection criteria “employed”. This definition was specified for some special cases. This specification has been described in detail earlier [25]. A total sample of 20,012 employees were included in the survey. This study was conducted as part of project no. F2456,^1^ conducted by the Federal Institute for Occupational Safety and Health (BAuA). For the current study, the sample was restricted to full-time workers (> 35 h/week) in the ages between 15 and under 67 years, resulting in a sample size of 14,414 participants. The resulting sample did not include minors under the age of 16 years. We employed a complete case analysis; therefore, a total of 14,331 persons with complete data were included in the study to examine the prevalence of MHL and its association to LBP (see Additional Table 1). As this is a secondary analysis of a sample with high number of participants, we did not perform a formal calculation of the sample size. Due to the large sample size, the power to detect even small difference between the categories of the exposure variable is high.

### Operationalization of the variables

All variables were based on self-reports of the participants and collected during the telephone interview of the 2018 BIBB/BAuA Employment Survey.

#### Outcome variable

During the telephone interview, participants were asked in German if they had experienced pain in their lower back in the last 12 months (“*Please tell me if you have had any of the following health problems during work or on working days in the past 12 months. We are interested in complaints that occurred frequently: Low back pain*” / author’s translation from German). Possible answers were “yes” or “no”. The question had been designed specifically for and applied similarly in all previous BIBB/BAuA employment surveys.

#### Exposure variable

To assess manual handling of heavy loads, participants were asked how often they had to lift heavy loads (> 20 kg for men and > 10 kg for women). Participants could answer “often”, “sometimes”, “rarely” or “never” to categorize how often they manually handled heavy loads.

#### Selected confounders

During the telephone interview, respondents were asked to answer questions on their gender (“men”, “women”), age (in years), actual weekly working hours, how often they stood, sat or worked in awkward postures (bending, kneeling, working overhead), climatic factors (“work in cold, heat, wet, damp or draught conditions”) and psychosocial workload (e.g. deadlines and performance pressure). The psychosocial index was operationalized as index (WL_PSY_) from 0 to 100 index points. The selection of items used in the index is comparable to the selection made by Kroll (2011) [28, 29]. Three subcategories (psychological stress, social burden, temporal involvement) were calculated by adding up points assigned to the items according to the answers. The achieved score was divided by the maximum possible points for valid answers. For WL_PSY_ the sum of these scores was standardized based on the total amount of validly answered subcategories. The higher the score of the index, the greater the psychosocial workload is assumed.

With the exception of awkward postures, working conditions were assessed in the same way as the manual handling of loads, using the categories “often”, “sometimes”, “rarely” and “never”. In the regression model, dummy variables were used to adjust for awkward postures. These were based on the questions regarding how often respondents worked in awkward postures (“often”, “sometimes”, “rarely” and “never”). Interviewers only asked for details on the specific type of posture (kneeling, working overhead, and working in bended postures) if participants responded “often”. Further information about the construction of the dummy variables is provided in the additional material (Additional Fig. 1). A German system for classifying occupations, published by Blossfeld, was used to assess occupational groups. This system divides occupations into 12 groups (agricultural occupations, unskilled manual occupations, skilled manual occupations, technicians, engineers, unskilled services, skilled services, semiprofessions, professions, unskilled commercial and administratorial occupations, skilled commercial and administratorial occupations, managers) [29].

### Missing data

The dataset of the 2018 BIBB/BAuA Employment Survey is only missing a small proportion of data. For the relevant exposure variable, only 0.1% of information is missing, and only 0.4% of information on outcome variable LBP. A complete case analysis was therefore performed, on the assumption that the data was missing completely at random.

### Statistical methods

The statistical analysis was performed fully syntax based using SPSS 25® statistical software. The description for all variables used are shown stratified by gender (“men”, “women”). For categorical variables, the absolute number (n) and relative frequencies have been presented and for numeric variables arithmetic mean, standard deviation (SD) and median have been provided. Unadjusted prevalences of MHL were estimated based on the descriptive statistics of the data of 14,331 participants. For gender-specific prevalences of MHL in different occupational groups, data was stratified separately for men and women based on the Blossfeld occupation code.

Associations between MHL and LBP were estimated using blockwise adjusted multivariate Poisson regression with robust variance estimates. This approach was used to obtain prevalence ratios (PR) directly from effect estimates of the model. 95% confidence intervals (95%CI) were calculated based on the robust variance estimates. PR with 95% confidence intervals were rounded to two decimal places and used as effect estimates of the association between MHL and LBP. We included sets of confounders step by step to receive information about their influence on the effect estimates resulting in five models. The models were built as follows:

- Unadjusted Model #0: only MHL as exposure, no other covariates
- Adjusted Model #1: unadjusted Model #0 + gender and age
- Adjusted Model #2: adjusted Model #1 + working hours
- Adjusted Model #3: adjusted Model #2 + further physical and climatic working conditions
- Main Model #4: adjusted Model #3 + psychosocial workload

In detail the main regression model was adjusted for gender, age, working hours, standing, sitting, awkward postures (bending, kneeling, working overhead), climatic factors and psychosocial workload. Confounders considered in the regression models were selected based on an a priori review of the available evidence of relevant cause-effect relationships. The resulting list was included in an underlying causal diagram and considered in the regression model [30]. The dependent variable was LBP. Postestimations were done on the basis of the main regression Model #4 to derive adjusted estimates of the prevalence of LBP, assuming that the cofactors are equally distributed. This leads to the assumption that the population exhibits an equally distributed proportion of men and women. To simplify the interpretation of the estimated prevalence of LBP age was centered to 45 years, working hours to 40 h/week (median of the subsample) and the index for WL_PSY_ to 38.9 points (arithmetic mean of the subsample). In the postestimation we controlled for these metric variables by choosing the value zero. Subsequently the results of the estimated prevalence refer to a person aged 45, who works 40 h/week, with a mean psychosocial workload of 38.9 index points. The resulting prevalence was rounded to one decimal place.

## Results

### Participants

In our analysis, 14,331 participants were included from the total dataset of the 2018 BIBB/BAuA Employment Survey (*n* = 20,012), of which 61.6% were men (*n* = 8828) and 38.4% women (*n* = 5503), with a median age of 49 (min = 16 years; max = 66 years). Participants (*n* = 5598) were not included if they did not meet the selection criteria of the study project F2456 of the BAuA. Of those *n* = 83 were not included in this specific analysis because they had missing data. For further information about missing data please see the additional material (Additional Table 1). On average respondents worked 43.81 h a week and indicated a psychosocial workload of 38.9 index points. Of this population, 52.8% of participants (*n* = 7570) said they were exposed to manual handling of heavy loads at work and 17.5% (*n* = 2505) said that they “often” manually handled loads during working hours. As to low back pain, 43.9% (*n* = 6294) stated that they had experienced pain in their lower back in the last 12 months. Further characteristics are provided in Table 1.

**Table 1:** Individual characteristics of the study population (*n* = 14,331, missings *n* = 0)

	Gender					
	Men		Women		Total	
	61.6% (*n* = 8828)		38.4% (*n* = 5503)		100% (*n* = 14,331)	
**Age in years**						
• *Mean*	46.2		47.5		46.7	
• *SD*	11.3		10.9		11.2	
• *Median*	48		50		49	
**Actual weekly working hours in h/week**						
• *Mean*	44.8		42.3		43.8	
• *SD*	7.8		7.0		7.6	
• *Median*	42		40		40	
**WL**_**PSY**_						
• *Mean*	39.1		38.5		38.9	
• *SD*	11.9		11.7		11.8	
• *Median*	37.7		37.2		37.4	
**Manual lifting of heavy loads**	**n**	**col%**	**n**	**col%**	**n**	**col%**
• *Never*	3884	44.0	2877	52.3	6761	47.2
• *Rarely*	2227	25.2	1033	18.8	3260	22.7
• *Sometimes*	1133	12.8	672	12.2	1805	12.6
• *Often*	1584	17.9^a^	921	16.7	2505	17.5
**Low back pain in the last 12 months**						
• *Yes*	3613	40.9	2681	48.7	6294	43.9
• *No*	5215	59.1	2822	51.3	8037	56.1
**Standing**						
• *Never*	1081	12.2	885	16.1	1966	13.7
• *Rarely*	1983	22.5	1167	21.2	3150	22.0
• *Sometimes*	1782	20.2	1047	19.0	2829	19.7
• *Often*	3982	45.1	2404	43.7	6386	44.6
**Sitting**						
• *Never*	1168	13.2	821	14.9	1989	13.9
• *Rarely*	1037	11.7	536	9.7	1573	11.0
• *Sometimes*	999	11.3	598	10.9	1597	11.1
• *Often*	5624	63.7^a^	3548	64.5	9172	64.0
**Bending**						
• *Yes*	781	8.8	468	8.5	1249	8,7
• *No*	8047	91.2	5035	91.5	13,082	91,3
**Kneeling**						
• *Yes*	7996	90.6	5063	92.0	13,059	91.1
• *No*	832	9.4	440	8.0	1272	8.9
**Working overhead**						
• *Yes*	8274	93.7	5338	97.0	13,612	95.0
• *No*	554	6.3	165	3.0	719	5.0
**Sometimes working in awkward postures**						
• *Yes*	1567	17.8	657	11.9	2224	15.5
• *No*	7261	82.2	4846	88.1	12,107	84.5
**Rarely working in awkward postures**						
• *Yes*	1293	14.6	590	10.7	1883	13.1
• *No*	7535	85.4	4913	89.3	12,448	86.9
**Climatic factors**						
• *Never*	4286	48.6	3611	65.6	7897	55.1
• *Rarely*	1301	14.7	594	10.8	1895	13.2
• *Sometimes*	1481	16.8	707	12.8	2188	15.3
• *Often*	1760	19.9	591	10.7^a^	2351	16.4

*SD* standard deviation, *n* absolute number of participants, *col%* column percentage; ^a^percentages do not count up to 100.0% due to rounding

### Prevalence of manual handling of heavy loads among different occupational groups

Regarding the prevalence of manual handling of heavy loads in different occupational groups (as defined by Blossfeld (1985)), the analysis shows that 56.1% (*n* = 105) of men and 54.8% (*n* = 34) of women in the agricultural sector answered “often”. For men, this is followed by persons working in skilled manual occupations (44.6%, *n* = 540), unskilled manual occupations (37.4%, *n* = 253) and unskilled services (31.2%, *n* = 271).

For women, the ranking continues with skilled manual occupations (39.3%, *n* = 64), unskilled manual occupations (34.4%, *n* = 53), semiprofessions (30.5%, *n* = 435) and unskilled services (30.2%, *n* = 67).

**Fig. 1:**
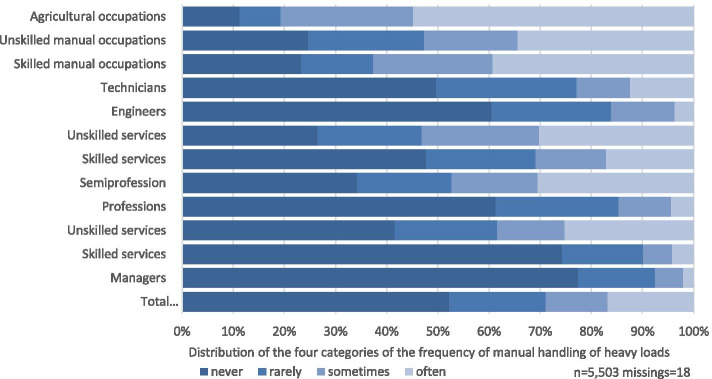
Self-reported frequency of MHL in women among different occupational groups

**Fig. 2:**
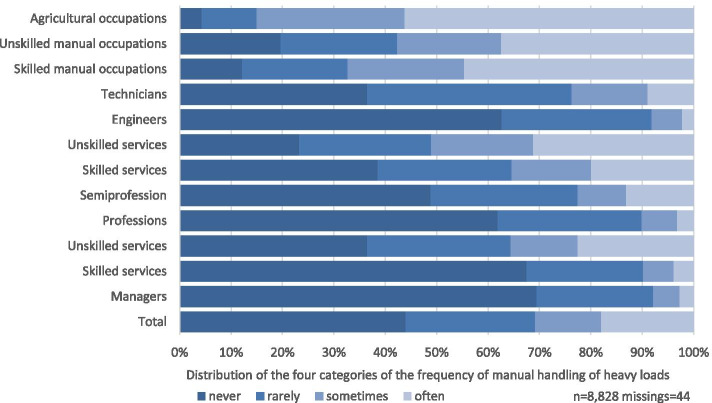
Self-reported frequency of MHL in men among different occupational groups

Women in semiprofessions answered “often” more frequently than men, with a total of 17.1 percentage points compared to 13.1% (*n* = 75) for men. Figures 1 and 2 show the prevalences of exposure to manual handling of heavy loads in the categories “never”, “rarely” and “sometimes” stratified by gender. The corresponding data is provided as Additional Table 2.

### Association between manual handling of heavy loads and low back pain

Table 2 shows the crude prevalence of low back pain as the outcome of interest, stratified by the self-reported frequency of manual handling of loads. The prevalence of low back pain increases from 36.0% (*n* = 2435) in participants who answered “never” to 65.9% (*n* = 1651) in participants who answered “often”. Furthermore, Table 2 shows the distribution of all considered confounders in the main Model #4, such as age, gender, working hours, and current weekly working time, physical and psychosocial workloads, stratified by the MHL categories.

**Table 2:** Manual handling of heavy loads stratified for important variables (*n* = 14,331; missings *n* = 0)

	Self-reported frequency of manual handling of loads (men > 20 kg and women > 10 kg)									
	Never		Rarely		Sometimes		Often		Total	
	*n*	*col%*	*n*	*col%*	*n*	*col%*	*n*	*col%*	*n*	*col%*
**Low back pain in the last 12 months**										
• *Yes*	2435	36.0	1311	40.2	897	49.7	1651	65.9	6294	43.9
• *No*	4326	64.0	1949	59.8	908	50.3	854	34.1	8037	56.1
**Gender**										
• *Men*	3884	57.4	2227	68.3	1133	62.8	1584	63.2	8828	61.6
• *Women*	2877	42.6	1033	31.7	672	37.2	921	36.8	5503	38.4
**Sitting**										
• *Often*	5575	82.5	2190	67.2	791	43.8	616	24.6	9172	64.0
• *Sometimes*	490	7.2	377	11.6	388	21.5	342	13.7	1597	11.1
• *Rarely*	273	4.0	401	12.3	255	14.1	644	25.7	1573	11.0
• *Never*	423	6.3	292	9.0^a^	371	20.6	903	36.0	1989	13.9
**Standing**										
• *Often*	1500	22.2	1429	43.8	1232	68.3	2225	88.8	6386	44.6
• *Sometimes*	1498	22.2	778	23.9	398	22.0	155	6.2	2829	19.7
• *Rarely*	2056	30.4	881	27.0	122	6.8	91	3.6	3150	22.0
• *Never*	1707	25.2	172	5.3	53	2.9	34	1.4	1966	13.7
**Bending**										
• *Often*	89	1.3	175	5.4	221	12.2	787	31.4	1272	8.9
• *Not often*	6672	98.7	3085	94.6	1584	87.8	1718	68.6	13,059	91.1
**Kneeling**										
• *Often*	89	1.3	175	5.4	221	12.2	787	31.4	1272	8.9
• *Not often*	6672	98.7	3085	94.6	1584	87.8	1718	68.6	13,059	91.1
**Working over head**										
• *Often*	36	0.5	94	2.9	118	6.5	471	18.8	719	5.0
• *Not often*	6725	99.5	3166	97.1	1687	93.5	2034	81.2.	13,612	95.0
**Sometimes working in awkward postures**										
• *Yes*	281	4.2	389	11.9	616	34.1	597	23.8	1883	13.1
• *No*	6480	95.8	2871	88.1	1189	65.9	1908	76.2	12,448	86.9
**Rarely working in an awkward postures**										
• *Yes*	429	6.3	992	30.4	377	20.9	426	17.0	2224	15.5
• *No*	6332	93.7	2268	69.6	1428	79.1	2079	83.0	12,107	84.5
**Climatic factors**										
• *Often*	330	4.9	428	13.1	421	23.3	1172	46.8	2351	16.4
• *Sometimes*	570	8.4	503	15.4	610	33.8	505	20.2	2188	15.3
• *Rarely*	505	7.5	876	26.9	249	13.8	265	10.6	1895	13.2
• *Never*	5356	79.2	1453	44.6	525	29.1	563	22.5^a^	7897	55.1
**Age in years**										
• *Mean*	47.24		47.06		45.55		45.33		46.65	
• *SD*	10.88		11.02		11.32		11.72		11.15	
• *Median*	49		49		48		48		49	
• *n*	6761		3260		1805		2505		14,331	
**Actual weekly working hours per week**										
• *Mean*	43.43		43.90		43.93		44.61		43.81	
• *SD*	6.79		7.66		8.57		8.71		7.60	
• *Median*	40		41		40		40		40	
**Psychosocial Workload Index WL**_**PSY**_ **(0-100)**										
• *Mean*	36.59		39.12		40.93		43.41		38.91	
• *SD*	10.83		11.52		12.13		13.02		11.84	
• *Median*	35.06		37.65		40.25		43.25		37.41	

*SD* standard deviation, *n* absolute number, *col%* column percentage; ^a^percentages do not count up to 100.0% due to rounding

### Association between MHL and LBP – results of the unadjusted model #0

In the unadjusted model, the prevalence ratio for LBP is estimated at 1.83 (95%CI [1.75; 1.91]) for participants who frequently handle heavy loads manually compared to participants who never handle heavy loads (“never”). In the categories “rare” (1.12 95%CI [1.06; 1.18]) and “sometimes” (1.38 95%CI [1.30; 1.46]) also reveal an association to manual handling of heavy loads compared to participants indicating no manual handling of heavy loads (“never”).

### Association between MHL and LBP – result of the main model #4 (adjusted)

**Fig. 3:**
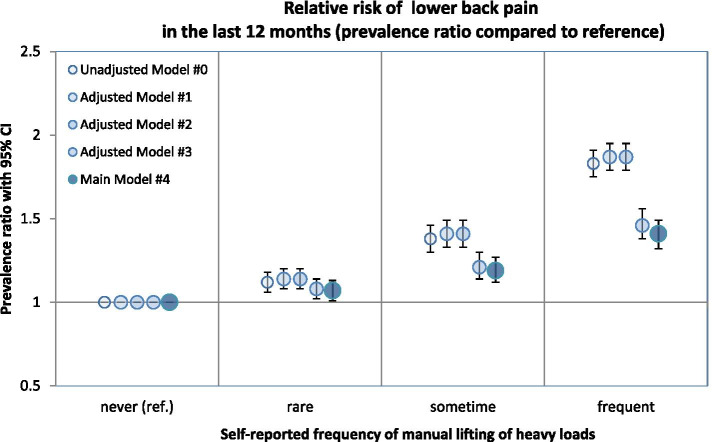
Relative risk of low back pain in the respond-categories of manual handling of heavy loads. Considered covariates in Model #4: age and gender, working hours, further physical and climatic working conditions and the psychosocial workload

After adjusting for gender, age, actual weekly working hours, and further working conditions, including psychosocial workload, this relationship decreases, as can be seen in Table 3. This decline was mainly due to the adjustment for further physical workloads (e.g. awkward postures) as can be seen in Fig. 3 with the decrease of the prevalence ratio from adjusted Model #2 to adjusted Model #3. Full results of the blockwise regression have been provided as additional material (Additional Table 3). Nevertheless, the positive association between the manual handling of heavy loads and LBP is revealed after adjusting for all selected confounders. The model-based, estimated prevalence of LBP increased with the frequency at which heavy loads were handled manually. Participants who said that they “often” handled heavy loads manually reported 40.7% more disorders in the lower back (PR 1.41 95%CI [1.32; 1.49]) compared to participants who said that they never handled loads manually (“never” MHL). Participants who said they “sometimes” (PR 1.19 95%CI [1.12; 1.27]) or “rarely” (1.07 95%CI [1.01; 1.13]) handled loads manually also showed an increased prevalence of LBP compared to the reference group (“never” exposed to MHL). The frequency of working in awkward postures (bending (PR 1.19 95%CI [1.11; 1.27]), kneeling (PR 1.11 95%CI [1.04; 1.19]), sometimes working in awkward postures (PR 1.10 95%CI [1.03; 1.16]), being exposed to hard climatic factors (PR: 1.22 95%CI [1.15; 1.28]), and increased psychosocial workload (PR: 1.11 95%CI [1.08; 1.13]; concerning a change of 10 index point) are also positively associated with LBP in the main Model #4. Furthermore, the results indicate that women (PR 1.21 95%CI [1.16; 1.25]) are at higher risk of low back pain. Sitting (PR 0.90 95%CI [0.85; 0.95]) during working hours and actual weekly working hours (PR 0.92 95%CI [0.90; 0.95], concerning a change of 10 h per week.) show a negative association with LBP. Table 3 shows the adjusted prevalence ratios of the main model.

**Table 3:** Association between MHL and LBP – results of the main model #4

	Adjusted prevalence ratios for self-reported low back pain in the last 12 month (PR (95%CI))
**Effect estimate of the association between MHL and LPB**	
**Manual handling of loads (exposure of interest)**	
• *Often*	1.41 (1.32; 1.50)
• *Sometimes*	1.19 (1.12;1.27)
• *Rarely*	1.07 (1.01; 1.13)
• *Never (reference)*	1
**Considered confounders in the main Model #4**	
**Gender**	
• *Women*	1.21 (1.17; 1.26)
• *Men (reference)*	1
**Sitting**	
• *Often*	0.90 (0.85; 0.95)
• *Sometimes*	0.83 (0.78; 0.89)
• *Rarely*	0.90 (0.85; 0.96)
• *Never (reference)*	1
**Standing**	
• *Often*	0.98 (0.91; 1.06)
• *Sometimes*	0.97 (0.90; 1.05)
• *Rarely*	1.01 (0.93; 1,08)
• *Never (reference)*	1
**Bending**	
• *Often*	1.19 (1.11; 1.27)
• *Not often (Reference)*	1
**Kneeling**	
• *Yes*	1.11 (1.04; 1.19)
• *No (reference)*	1
**Working over head**	
• *Yes*	0.98 (0.91; 1.05)
• *No (reference)*	1
**Sometimes working in awkward postures**	
• *Yes*	1.10 (1.03; 1.16)
• *No (reference)*	1
**Rarely working in an awkward postures**	
• *Yes*	1.01 (0.95; 1.07)
• *No (reference)*	1
**Climatic factors**	
• *Often*	1.22 (1.15; 1.28)
• *Sometimes*	1.14 (1.08; 1.20)
• *Rarely*	1.03 (0.97; 1.09)
• *Never (reference)*	1
**Numeric parameters**	
• *Age (per 10 years)*	1.06 (1.04; 1.07)
• *Working hours (per 10 h per week)*	0.92 (0.90; 0.95)
• *Psychosocial working conditions (per 10 points)*	1.11 (1.08; 1.13)

*95%CI* 95% confidence interval

### Estimated prevalences of low back pain in the main model #4

The estimates of the prevalence of LBP based on the adjusted model (main Model #4) increase with the frequency at which heavy loads are handled manually. 47.3% (95%CI [43.8%; 51.1%]) of participants who said they never handle heavy loads manually (“never”) indicated complaints in the low back. In the “rarely” category, this value increases to 50.4% (95%CI [46.8%; 54.3%]), and for participants who answered that they “sometimes” handle loads manually it is estimated to be 56.4% (95%CI [52.4%; 60.6%]). For those who answered that they “often” lift heavy loads manually, the estimated percentage of persons who reported pain in their lower back rises to 66.5% (95%CI [62.4%; 71.0%]).

## Discussion

The aim of the study was to determine the current prevalence of MHL in different occupational groups stratified by gender in Germany, the association between this physical strain and low back pain and to estimate adjusted prevalence of LPB in the respond categories of MHL.

The analysis of the German Employment Survey of the Working Population on Qualification and Working Conditions reveals that employees in the agricultural sector frequently handle loads manually (“often”), with a prevalence of over 50% for both men and women. Persons employed in skilled and unskilled manual occupations or unskilled services also indicate a high prevalence of manual handling of heavy loads. Women who work in semiprofessions are also often exposed to the manual handling of heavy loads at work, with 30.5% responding that they “often” manually handle heavy loads. Only 13.1% of men working in these professions said they were “often” exposed to these activities at work. These results correspond to other studies on Germany’s working population, like the so called “DGB Report” (grey literature) provided by German Trade Union, which reports that 55% of employees in Germany perform heavy physical work (including manual handling of heavy loads) [1, 31]. A comparison of these results is however difficult, because of the different settings of the studies. Although data about manual handling of heavy loads were assessed in previous BIBB/BAuA Employment Surveys an evaluation of a change of exposure prevalence is difficult to interpret because participants differ in the surveys, there might be a change in the occupational structure and the overall settings of the surveys.

Furthermore, it is a well-known fact that manual handling of loads is a risk factor for musculoskeletal diseases in persons who work in the agricultural sector [32, 33].

The analysis of this survey underlines the well-known relationship between manual lifting or carrying of heavy loads and LBP [5, 6]. After adjusting for gender, age, actual weekly working hours and physical, climatic and psychosocial working conditions, the prevalence of LBP in employees who frequently handle heavy loads manually is 1.41 times higher than in employees who are not exposed to such activities. According to other studies, effect estimates vary between 1.5 and 3.1, depending on selected confounders [6]. Due to the subjective measurement of the exposition in this study, it is possible that the estimated effect is too low [6]. This may be a reason for the lower effect found in this analysis.

When the adjusted prevalence of LBP for workers who frequently handle heavy loads manually is compared to workers who don’t handle heavy loads, it is shown that 19.2 percentage points of LBP can be avoided by reducing manual handling of heavy loads. This underlines the huge potential for primary prevention of MHL to reduce the prevalence of LBP in workers.

Compared to the unadjusted model the effect decreases with the adjustment for the selected covariables. According to the results of the blockwise regression, the main reason for the reduction seems to be the adjustment for physical and climatic working conditions (sitting, standing, awkward postures and climatic factors). The difference between the results of the unadjusted and adjusted model emphasizes the importance of adjustment, when analysing physical strains.

With the increasing frequency of MHL, the estimated prevalences of LBP rise from 47.3% for employees who responded that they do not handle heavy loads manually to 66.5% for those who answered that they “often” handle heavy loads manually. It is already known that the frequency of MHL has an impact on the mechanical strain of the spine [6]. The results support this knowledge.

The results of this analysis of the 2018 BIBB/BAuA Employment Survey contribute to the third period of the Joint German Occupational Safety and Health Strategy (“GDA”, www.gda-portal.de), in which specific workplace preventions programs are put into practice to minimise the impact of physical workloads. This supports also the need for prevention of work-related musculoskeletal disorders aimed in the current EU-OSHA campaign “Lighten the Load” 2020-22 (https://healthy-workplaces.eu/en).

Some limitations of the study should be considered. The data originated from a cross-sectional study and was collected via telephone. For this reason, there may be selection bias due to the respondents’ availability by phone and their willingness to participate (“self-selection bias”). Efforts were made to obtain the basic data of the non-responders by means of a short questionnaire. However, response rates were too low to allow a comparison of the groups. Interviews were conducted in German; this is why persons without an adequate command of the language have been excluded. Therefore, important data of a potentially vulnerable group of employees is missing.

All variables are based on self-reports of the participants, which leads to an information bias due to memory failure and recall bias. With regard to the latter, it is known that persons with complaints tend to overestimate exposure; in other words, persons without complaints underreport exposure [34]. The resulting differential misclassification of the exposure status can lead to an overestimation of the effect [34]. Recall bias may also result in respondents underestimating the effect [34]. However, objective measurements cannot be obtained for the high number of participants (*n* = 20,012) analysed in this study.

It should be kept in mind, that the major part of the questions of the BIBB/BAuA Employment Survey are self-developed items, which cannot be linked to validated questionnaires [27]. For instance, LBP was only assessed by asking if it occurred; how often the pain occurred and how intense it was remains unclear. If the question regarding LBP was affirmed, the case definition in this study was fulfilled. This case definition is not in line with a consensus- and synthesised-based case definition for non-specific LBP by van der Molen et al. (2021) [35], who summarized previous case definition applied in the literature.

The assessment of MHL in the categories “often”, “sometimes”, “rarely” and “never” based on a pre-set specification of heavy loads as weights greater or equal than 20 kg for men and 10 kg for women could have caused an underestimation of the effect size regarding manual handling of loads. It should be mentioned that according to Kuijer et al. (2014) [36] a risk assessment for loads between 3 and 25 kg is advised. Therefore, it is possible, that persons who reported no manual handling of heavy loads could have pain in their lower back due to manual handling of loads with less weight than indicated in the question in the survey. As this might have affected our reference group, we cannot exclude a possible underestimation of the effect size.

It should be kept in mind, that physical work exposures do not occur isolated in real life. For example, results of the analysis of the 2018 BIBB/BAuA Employment Survey show, that 61.5% of persons, who frequently work in awkward postures and 41.1% of persons, who perform manual handling operations also reported frequent manual handling of heavy loads [37, 38]. Andersen et al. (2021) [39] underline the importance of combined physical workload in the development of MSD. The results of their prospective cohort study, which compared clusters with different exposure combinations, showed that workers exposed to a combination of lifting/carrying, pushing/pulling, working in awkward postures for most of their working time and performing manual handling operations had the largest increase in pain in their lower back [39].

In addition, the question on the assessment of climatic factors in particular is imprecise (see Operationalization). The index used for the operationalization of psychosocial workload is based on the calculation of a validated index. Secondary data was used to investigate the research questions. The 2018 German Employment Survey of the Working Population on Qualification and Working Conditions does not provide all confounders that are considered important for the investigated research question. For instance, smoking status, anthropometric data and occupational exposure to whole-body-vibrations were not available in the dataset and could not be included in the analysis. However, there may be over-adjustment bias, as a high number of cofactors that were included as models were built based on knowledge obtained from the literature [40, 41].

When interpreting the results, it should be kept in mind that the data exclusively originated from employed persons, who tend to be healthier in comparison to the total population. This can lead to bias due to the healthy worker effect. Therefore, the results cannot be applied in full to the general situation in Germany. The weighted sample of the 2018 BiBB/BAuA Survey with *n* = 20,012 participants is representative for the German labour force. The weighting factor included age, gender, household size, marital status, job position, nationality and state of residence. However, in this analysis we used a subsample, which was restricted to age, and weekly working hours. Therefore, we could not apply the weighting factor to our subsample. Consequently, the representativeness of our subsample is limited.

Although this cross-sectional study cannot reveal a causal relationship, the dataset represents the largest survey that considers the working conditions of the German working population with an absolute number of 20,012. Furthermore, the aim of the study was to derive prevalence of MHL in the work force in Germany. By using a dataset with such a high number of participants, it is possible to detect even small effects between the exposure categories regarding the outcome. Statistical significance of small differences alone may not be relevant, and do not implicate a need for a practical intervention or prevention approach.

The 2018 BIBB/BAuA Employment Survey could be used to generate a Job Exposure Matrix [42], because it provides a huge dataset with information about wide range of work-related exposures and occupational groups.

## Conclusion

It is still a common occurrence for the German work force to be exposed to the manual handling of heavy loads at work, even though this is already known to be a risk factor, particularly for the lower back. Furthermore, we could confirm an association between manual handling of heavy loads and lower back pain in this secondary analysis. The surveillance of physical strains remains important. According to the results of this study, avoiding this physical workload has huge potential to prevent pain in the lower back – especially in professions in the agricultural sector (i.e. farmers), unskilled manual occupations (i.e. construction helpers), skilled manual occupations (i.e. locksmiths) and in unskilled services (i.e. waiters) –and should furthermore be sustained.

## Supplementary Information

**Additional file 1: Additional Table 1.** Number of missing values per item after applying of selection criteria considering participants aged < 67 and at least 35 h weekly working time. Number of missing values per item used in the main Model #4 after applying of selection criteria considering participants aged < 67 and at least 35 h weekly working time.

**Additional file 2: Additional Table 2.** Manual handling of heavy loads in occupational groups (*n* = 14,331; missings *n* = 62). The table present the data of manual handling of heavy loads in different occupational groups in men and women. Data was presented as figure in the manuscript.

**Additional file 3: Additional Table 3.** Prevalence ratios of further models. Models used in the blockwise regression analyses and main results of the models regarding the relative risk of low back pain in the last 12 months stratified by the self-reported frequency of manual handling of heavy loads.

**Additional file 4: Additional Figure 1.** Construction of the dummy-variables. Flowchart of the construction of the dummy-variables for working in awkward postures used in the regression analyses.

## Data Availability

The dataset (number ZA7574) supporting the conclusions of this article is available as scientific-Use-File. The access to the dataset is closed. Administrative permission to access and use can be requested externally at „BIBB – Bundesinstitut für Berufsbildung - Forschungsdatenzentrum “(Postfach 201264; 53142 Bonn; Germany; fax number: + 49 – (0)228 – 107 – 2020)). The dataset will be available as ftp-download after approved application.

## Abbreviations

EU-OSHA: European Agency of Safety and Health at Work
LPB: Low back pain
MHL: Manual handling of heavy loads
PR: Prevalence ratio
WL_PSY_: Index for psychosocial workload

## Footnotes

1 www.baua.de/DE/Aufgaben/Forschung/Forschungsprojekte/f2456.html
