# Supplementary material for: Manual handling of heavy loads and low back pain among different occupational groups: results of the 2018 BIBB/BAuA employment survey
Source: BMC Musculoskelet Disord. 2021 Nov 15;22:956. doi: 10.1186/s12891-021-04819-z (PMC8594139; doi:10.1186/s12891-021-04819-z)
Supplement: Supplementary file 1 — Additional file 1: Additional Table 1. Number of missing values per item after applying of selection criteria considering participants aged < 67 and at least 35 h weekly working time. Number of missing values per item used in the main Model #4 after applying of selection criteria considering participants aged < 67 and at least 35 h weekly working time. [file 12891_2021_4819_MOESM1_ESM.docx]

Additional Table 1: Number of missing values per item after applying of selection criteria considering participants aged <67 and at least 35 hours weekly working time

| Variable considered in the  complete case analysis | Number of  participants available | Missing  values per item | Remaining subjects with valid items |
| --- | --- | --- | --- |
| Items used as selection criteria: |  |  |  |
| Age (<67 years) | 14,414 | 0 | 14,414 |
| Weekly working hours (>= 35h) | 14,414 | 0 | 14,414 |
| Other items: |  |  |  |
| Lower back pain | 14,414 | 26 | 14,388 |
| Manual handling of heavy loads | 14,414 | 6 | 14,408 |
| Gender | 14,414 | 0 | 14,414 |
| Sitting | 14,414 | 21 | 14,393 |
| Standing | 14,414 | 25 | 14,389 |
| Working in awkward postures | 14,414 | 10 | 14,404 |
| Climatic factors (“Cold; heat;  wet humidity; draught”) | 14,414 | 14 | 14,414 |
| Psychosocial working conditions | 14,414 | 25 | 14,389 |
| Total | 14,414 cases  available after application of the selection  criteria | 83 cases  with missing values in one or more  variables | 14,331 complete cases  used in the complete case analysis |
