# Supplementary material for: Manual handling of heavy loads and low back pain among different occupational groups: results of the 2018 BIBB/BAuA employment survey
Source: BMC Musculoskelet Disord. 2021 Nov 15;22:956. doi: 10.1186/s12891-021-04819-z (PMC8594139; doi:10.1186/s12891-021-04819-z)
Supplement: Supplementary file 2 — Additional file 2: Additional Table 2. Manual handling of heavy loads in occupational groups (n = 14,331; missings n = 62). The table present the data of manual handling of heavy loads in different occupational groups in men and women. Data was presented as figure in the manuscript. [file 12891_2021_4819_MOESM2_ESM.docx]

Additional Table 2: Manual handling of heavy loads in occupational groups (n=14,331; missings n=62)

|  | Self-reported frequency of manual handling of heavy loads  (men > 20 kg and women > 10 kg) | | | | | | | | | |
| --- | --- | --- | --- | --- | --- | --- | --- | --- | --- | --- |
| Blossfeld’s  occupational group | **Never** | | **Rarely** | | **Sometimes** | | **Often** | | **Total** | |
|  | n | row% | n | row% | n | row% | n | row% | n | row% |
| Men (n=8,828, missings n=44) | | | | | | | | | | |
| Agricultural occupations | 8 | 4.3 | 20 | 10.7 | 54 | 28.9 | 105 | 56.1 | 187 | 100.0 |
| Unskilled  manual  occupations | 133 | 19.7 | 153 | 22.6 | 137 | 20.3 | 253 | 37.4 | 676 | 100.0 |
| Skilled manual occupations | 148 | 12.2 | 248 | 20.5 | 274 | 22.6 | 540 | 44.6 | 1,210 | 99.9* |
| Technicians | 228 | 36.5 | 249 | 39.8 | 92 | 14.7 | 56 | 9.0 | 625 | 100.0 |
| Engineers | 483 | 62.7 | 224 | 29.1 | 46 | 6.0 | 17 | 2.2 | 770 | 100.0 |
| Unskilled  services | 202 | 23.3 | 223 | 25.7 | 172 | 19.8 | 271 | 31.2 | 868 | 100.0 |
| Skilled  services | 211 | 38.5 | 143 | 26.1 | 85 | 15.5 | 109 | 19.9 | 548 | 100.0 |
| Semi- professions | 280 | 48.9 | 164 | 28.6 | 54 | 9.4 | 75 | 13.1 | 573 | 100.0 |
| Professions | 330 | 61.9 | 149 | 28.0 | 37 | 6.9 | 17 | 3.2 | 533 | 100.0 |
| Unskilled commercial and administratorial occupations | 81 | 36.5 | 62 | 27.9 | 29 | 13.1 | 50 | 22.5 | 222 | 100.0 |
| Skilled commercial and administra- torial occupations | 1063 | 67.6 | 356 | 22.6 | 94 | 6.0 | 60 | 3.8 | 1573 | 100.0 |
| Managers | 694 | 69.5 | 227 | 22.7 | 51 | 5.1 | 27 | 2.7 | 999 | 100.0 |
| Total | 3861 | 44.0 | 2218 | 25.3 | 1125 | 12.8 | 1580 | 18.0 | 8784 | 100.1* |
| Women (n=5,503, missings=18) | | | | | | | | | | |
| Agricultural occupations | 7 | 11.3 | 5 | 8.1 | 16 | 25.8 | 34 | 54.8 | 62 | 100.0 |
| Unskilled  manual  occupations | 38 | 24.7 | 35 | 22.7 | 28 | 18.2 | 53 | 34.4 | 154 | 100.0 |
| Skilled  manual  occupations | 38 | 23.3 | 23 | 14.1 | 38 | 23.3 | 64 | 39.3 | 163 | 100.0 |
| Technicians | 96 | 49.7 | 53 | 27.5 | 20 | 10.4 | 24 | 12.4 | 193 | 100.0 |
| Engineers | 83 | 60.6 | 32 | 23.4 | 17 | 12.4 | 5 | 3.6 | 137 | 100.0 |
| Unskilled  services | 59 | 26.6 | 45 | 20.3 | 51 | 23.0 | 67 | 30.2 | 222 | 100.1* |
| Skilled  services | 221 | 47.7 | 99 | 21.4 | 64 | 13.8 | 79 | 17.1 | 463 | 100.0 |
| Semi- professions | 488 | 34.2 | 264 | 18.5 | 239 | 16.8 | 435 | 30.5 | 1426 | 100.0 |
| Professions | 324 | 61.4 | 127 | 24.1 | 54 | 10.2 | 23 | 4.4 | 528 | 100.1* |
| Unskilled commercial and administratorial occupations | 114 | 41.6 | 55 | 20.1 | 36 | 13.1 | 69 | 25.2 | 274 | 100.0 |
| Skilled commercial and administratorial occupations | 989 | 74.3 | 211 | 15.9 | 76 | 5.7 | 55 | 4.1 | 1331 | 100.0 |
| Managers | 412 | 77.4 | 80 | 15.0 | 29 | 5.5 | 11 | 2.1 | 532 | 100.0 |
| Total | 2869 | 52.3 | 1,029 | 18.8 | 668 | 12.2 | 919 | 16.8 | 5485 | 100.1* |

Legend: n: absolute number of participants, row%: row percentage; *percentages do not count up to 100.0% due to rounding.
