# Supplementary material for: Manual handling of heavy loads and low back pain among different occupational groups: results of the 2018 BIBB/BAuA employment survey
Source: BMC Musculoskelet Disord. 2021 Nov 15;22:956. doi: 10.1186/s12891-021-04819-z (PMC8594139; doi:10.1186/s12891-021-04819-z)
Supplement: Supplementary file 3 — Additional file 3: Additional Table 3. Prevalence ratios of further models. Models used in the blockwise regression analyses and main results of the models regarding the relative risk of low back pain in the last 12 months stratified by the self-reported frequency of manual handling of heavy loads. [file 12891_2021_4819_MOESM3_ESM.docx]

Additional Table 3: Prevalence ratios for low back pain of the models used in the blockwise regression

| Regression models  (included covariables) | Self-reported frequency of manual lifting of heavy loads  (men > 20 kg and women > 10 kg) | | | |
| --- | --- | --- | --- | --- |
|  | Never (reference) | Rarely | Sometimes | Often |
| Unadjusted Model #0 | 1 | 1.12 (1.06; 1.18) | 1.38 (1.31; 1.46) | 1.83 (1.76; 1.91) |
| Adjusted Model #1  *(including gender, age)* | 1 | 1.14 (1.08; 1.20) | 1.41 (1.33; 1.49) | 1.87 (1.79; 1.95) |
| Adjusted Model #2  *(including gender, age, working hours)* | 1 | 1.14 (1.09; 1.20) | 1.41 (1.33; 1.49) | 1.87 (1.79; 1.95) |
| Adjusted Model #3 *(including gender, age, working hours, further physical and climatic working conditions)* | 1 | 1.08 (1.02; 1.14) | 1.21 (1.14; 1.30) | 1.46 (1.38; 1.56) |
| Main Model #4 *(including gender, age, working hours, further physical and climatic working conditions,  psychosocial workload*) | 1 | 1.07 (1.01; 1.13) | 1.19 (1.12; 1.27) | 1.41 (1.32; 1.49) |
| Centering of confounder variables: age=45 years; working time=40 h per week, psychosocial working conditions=38.9 index points. Further cofactors are equally distributed.  Prevalence ratios with 95 % confidence limits. | | | | |
