# Supplementary material for: Manual handling of heavy loads and low back pain among different occupational groups: results of the 2018 BIBB/BAuA employment survey
Source: BMC Musculoskelet Disord. 2021 Nov 15;22:956. doi: 10.1186/s12891-021-04819-z (PMC8594139; doi:10.1186/s12891-021-04819-z)
Supplement: Supplementary file 4 — Additional file 4: Additional Figure 1. Construction of the dummy-variables. Flowchart of the construction of the dummy-variables for working in awkward postures used in the regression analyses. [file 12891_2021_4819_MOESM4_ESM.docx]

Additional Figure 1: Working in awkward postures: Construction of the dummy-variables used in the regression analyses
